# Impact of different metal artifact reduction techniques in photon-counting computed tomography head and neck scans in patients with dental hardware

**DOI:** 10.1007/s00330-023-10430-8

**Published:** 2023-11-16

**Authors:** Fabian Bernhard Pallasch, Alexander Rau, Marco Reisert, Stephan Rau, Thierno Diallo, Thomas Stein, Sebastian Faby, Fabian Bamberg, Jakob Weiss

**Affiliations:** 1 Department of Radiology, University Medical Center Freiburg, Hugstetter Str. 55, 79106 Freiburg im Breisgau, Germany; 2 Siemens Healthcare GmbH, Siemensstr. 3, 91301 Forchheim, Germany

**Keywords:** Multidetector computed tomography, Artifacts, Dental implants, Image processing (Computer-assisted), Head and neck neoplasms

## Abstract

**Objectives:**

Metal artifacts remain a challenge in computed tomography. We investigated the potential of photon-counting computed tomography (PCD-CT) for metal artifact reduction using an iterative metal artifact reduction (iMAR) algorithm alone and in combination with high keV monoenergetic images (140 keV) in patients with dental hardware.

**Material and methods:**

Consecutive patients with dental implants were prospectively included in this study and received PCD-CT imaging of the craniofacial area. Four series were reconstructed (standard [PCD-CT_std_], monoenergetic at 140 keV [PCD-CT_140keV_], iMAR corrected [PCD-CT_iMAR_], combination of iMAR and 140 keV monoenergetic [PCD-CT_iMAR+140keV_]). All reconstructions were assessed qualitatively by four radiologists (independent and blinded reading on a 5-point Likert scale [5 = excellent; no artifact]) regarding overall image quality, artifact severity, and delineation of adjacent and distant anatomy. To assess signal homogeneity and evaluate the magnitude of artifact reduction, we performed quantitative measures of coefficient of variation (CV) and a region of interest (ROI)–based relative change in artifact reduction [PCD-CT/PCD-CT_std_].

**Results:**

We enrolled 48 patients (mean age 66.5 ± 11.2 years, 50% (*n* = 24) males; mean BMI 25.2 ± 4.7 kg/m^2^; mean CTDI_vol_ 6.2 ± 6 mGy). We found improved overall image quality, reduced artifacts and superior delineation of both adjacent and distant anatomy for the iMAR vs. non-iMAR reconstructions (all *p* < 0.001). No significant effect of the different artifact reduction approaches on CV was observed (*p* = 0.42). The ROI-based analysis indicated the most effective artifact reduction for the iMAR reconstructions, which was significantly higher compared to PCD-CT_140keV_ (*p* < 0.001).

**Conclusion:**

PCD-CT offers highly effective approaches for metal artifact reduction with the potential to overcome current diagnostic challenges in patients with dental implants.

**Clinical relevance statement:**

Metallic artifacts pose a significant challenge in CT imaging, potentially leading to missed findings. Our study shows that PCD-CT with iMAR post-processing reduces artifacts, improves image quality, and can possibly reveal pathologies previously obscured by artifacts, without additional dose application.

**Key Points:**

• *Photon-counting detector CT (PCD-CT) offers highly effective approaches for metal artifact reduction in patients with dental fillings/implants*.

• *Iterative metal artifact reduction (iMAR) is superior to high keV monoenergetic reconstructions at 140 keV for artifact reduction and provides higher image quality*.

• *Signal homogeneity of the reconstructed images is not affected by the different artifact reduction techniques*.

## Introduction

Artifacts caused by metallic implants constitute a major challenge in computed tomography (CT) imaging and their prevalence is expected to further increase due to demographic changes in Western societies [1, 2]. The underlying physical principles causing these artifacts include photon starvation [3], photon scattering, and beam hardening [4], resulting in streaks and shadows adjacent to the implants [5] with the risk of missing potentially relevant findings (e.g., abscesses, fractures, or malignancies) [6].

To overcome these limitations, several strategies for artifact reduction have been proposed over the last decades including iterative metal artifact reduction (iMAR) techniques [7], reconstruction of virtual monoenergetic imaging at high keV [8, 9], and sinogram inpainting approaches [10], which have shown promising results in different scenarios such as dental hardware and orthopedic implants and prostheses [6, 8, 11, 12].

With the recent introduction of photon-counting detector CT (PCD-CT), a new opportunity for artifact reduction has become available [13]. In contrast to conventional energy-integrating detectors (EID) that convert the incoming photons into electric current via visible light [14], photon-counting detectors facilitate a direct conversion with the potential to count individual photons and measure their energy level within the detected spectrum [14]. This spectral information acquired in every scan allows for advanced and more accurate post-processing (including virtual monoenergetic and iterative reconstruction techniques) then conventional EID-CT data and has shown encouraging results in first clinical applications [15–19].

Thus, we explored the potential of an iMAR algorithm alone and in combination with high keV monoenergetic images at 140 keV on a first-generation clinical PCD-CT for metal artifact reduction in patients with dental implants and restorations. We hypothesized that these different approaches with the novel PCD-CT technology allow for efficient artifact reduction resulting in significantly improved subjective and objective image quality.

## Material and methods

### Patient population

The study was approved by the local ethics committee and all participants provided written consent. No examination was performed for study purposes only. In this prospective single-center study, consecutive patients from July 2022 to September 2022 with dental restorations and/or non-removable metallic implants underwent contrast-enhanced PCD-CT imaging for routine clinical workup. Inclusion criteria were clinical indication for craniofacial CT imaging with known pathologies potentially affecting the oral cavity or neck (e.g., oropharyngeal carcinoma, abscesses, lymph node metastasis) and the presence of dental hardware. Exclusion criteria were contraindications for CT imaging (allergy to iodine contrast agent, renal impairment, and thyroid dysfunction) and patients younger than 18 years.

### CT protocol and image reconstruction

All CT examinations were performed on a first-generation clinical dual source PCD-CT system (NAEOTOM Alpha, Siemens Healthineers) in a supine position. Images were acquired in portal-venous phase 75 s after body weight adapted contrast agent administration (1.2 mg/kg; flow 2 mL/s) using a dual-syringe power injector followed by a 20-mL saline flush. Acquisition parameters were as follows: multispectral mode with CAREDose4D and CARE keV enabled; CARE keV IQ level = 145 (corresponding to a ref mAs ranging from 55 to 100 depending on examination protocol) in Quantumplusmode (120 kV or 140 kV), collimation 144 × 0.40 mm; pitch = 0.8; gantry rotation time = 0.25 s.

Subsequently, four axial series were reconstructed from the acquired raw data:

1. Standard reconstruction (PCD-CT_std_): soft tissue (Br40) kernel, quantum iterative reconstruction (strength 4), slice thickness of 3 mm, increment of 3 mm, monoenergetic energy level of 60 keV as per our institutional standard.
2. Virtual monoenergetic high keV reconstructions (PCD-CT_140keV_): virtual monoenergetic energy level of 140 keV as previously demonstrated by Anhaus et al 2022 [20] and the same reconstruction parameters as in (1).
3. Iterative metal artifact reduction reconstruction (PCD-CT_iMAR_; iMAR): using a iMAR algorithm (Siemens Healthineers) and the same reconstruction parameters as in (1). The iMAR algorithm combines in an iterative loop the following types of corrections: a beam hardening correction and a normalized sinogram inpainting in raw data space, as well as a frequency split in image domain [21, 22]. The algorithm is an adapted solution of the previously introduced version developed for EID-CT, which uses a different parametrization and thresholds optimized for the PCD-CT imaging spectra.
4. A reconstruction combining the iMAR algorithm and the 140 keV monoenergetic reconstruction (PCD-CT_iMAR + 140keV_) with similar reconstruction parameters as in (1).

### Qualitative image analysis

**Fig 1:**
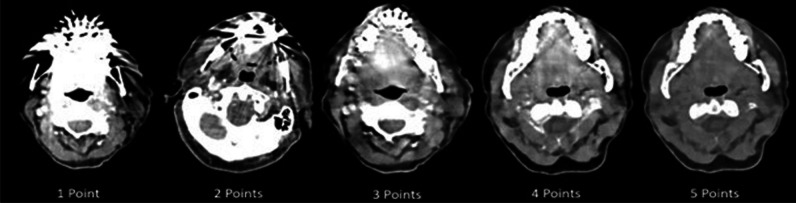
Example of subjective image quality analysis using a 5-point Likert scale for overall image quality, artifact severity, and delineation of adjacent as well as distant anatomy, which were rated as follows: 1 = non-diagnostic; 2 = poor, severe artifacts; 3 = fair, moderate artifacts; 4 = good, minor artifacts; 5 = excellent, no artifacts

All reconstructions were qualitatively assessed by four radiologists (1^st^, 3^rd^, 5^th^ year residents, and an experienced attending physician) independently using a 5-point Likert scale in a random fashion and blinded to the type of reconstruction. All readings were performed on a dedicated workstation using a research image processing software (nora; https://www.nora-imaging.com/) with respect to the following criteria: (i) overall image quality, (ii) artifact severity, (iii) delineation of adjacent anatomy (defined as malignancies, lymph nodes, abscesses, bone fractures), and (iv) delineation of distant anatomy (defined as spinal musculature, carotid artery and jugular vein, lymph nodes), which were rated as follows: 5 = excellent, no artifacts; 4 = good, minor artifacts; 3 = fair, moderate artifacts; 2 = poor, severe artifacts; 1 = non-diagnostic (see Fig. 1).

### Quantitative image analysis

#### Coefficient of varication (CV)

To investigate whether the different artifact reduction approaches affect or alter the homogeneity of the image signal, we calculated the CV by placing a region of interest (ROI; 150 mm^2^) in muscle tissue that was subjectively unaffected by the artifact. To ensure an accurate comparison, the size and localization of the ROI were kept constant between the different reconstructions (PCD-CT_std_, PCD-CT_140keV_, PCD-CT_iMAR_, PCD-CT_iMAR+140keV_). From the ROI, the mean Hounsfield units (HU) and standard deviation (SD) were extracted to calculate the CV as follows:

$$\mathrm{CV }= {\mathrm{SD}}_{\mathrm{ROI}} / {\mathrm{HU}}_{\mathrm{ROI}}$$

#### Quantification of artifact reduction

**Fig 2:**
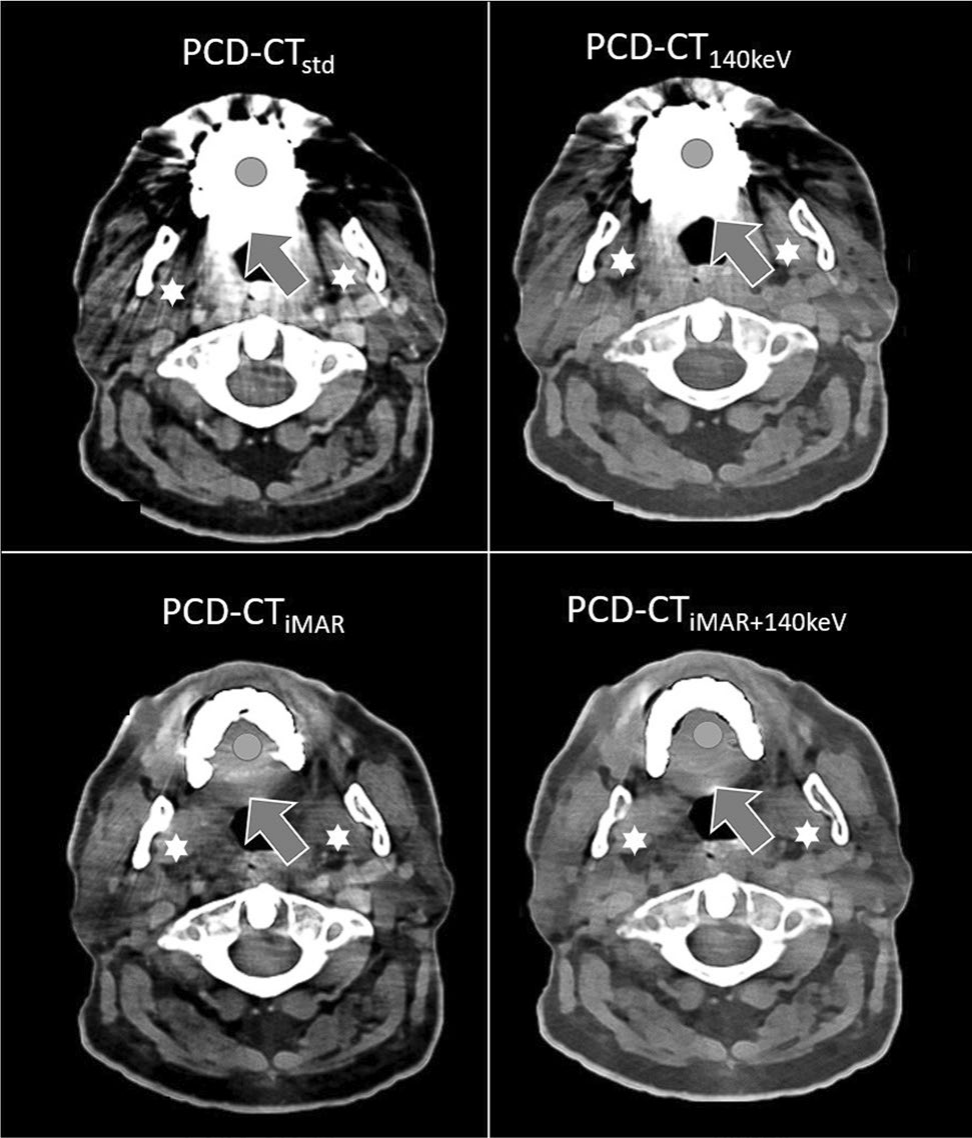
Image example of a 67-year-old patient with lymphoma. Evaluation of the root of the tongue (arrow) as well as the submandibular glands (stars) is substantially improved in PCD-CT_iMAR_ and PCD-CT_iMAR+140keV_ reconstructions compared to PCD-CT_std_ and PCD-CT_140keV_ only reconstructions. The circle represents the ROI to compare the artifact reduction potential of the different approaches based on relative changes in Hounsfield units. PCD-CT_std_, standard reconstruction without metal artifact reduction; PCD-CT_140keV_, virtual monoenergetic reconstruction at 140 keV; PCD-CT_iMAR_, iterative metal artifact reduction algorithm; PCD-CT_iMAR+140keV_, iterative metal artifact reduction algorithm in combination with virtual monoenergetic reconstruction at 140 keV

To objectively quantify the artifact reduction potential of the different investigated artifact reduction approaches, we estimated the change in artifact severity based on relative changes in HU within the artifact. First, a single image at the level with the subjectively most severe artifact was defined (by FP) on PCD-CT_std_ for all patients. Subsequently, the corresponding images in PCD-CT_140keV_, PCD-CT_iMAR_, and PCD-CT_iMAR+140keV_ reconstructions were identified. Thereafter, the mean HU values were measured on all reconstructions within the artifact using a circular ROI (150 mm^2^) (Fig. 2). Finally, the relative change in HU between the different artifact reduction techniques was calculated as follows with PCD-CT_std_ serving as reference:

$$\mathrm{Quotient }= (\mathrm{PCD}-\mathrm{CT }/\mathrm{ PCD}-{\mathrm{CT}}_{\mathrm{std}}) * 100$$

The output of this calculation represents an objective change in image signal that can be attributed to the artifact reduction technique applied and was interpreted as a quantitative measure of artifact reduction severity.

### Statistical analysis

R Foundation for Statistical Computing was used for statistical analyses. Continuous variables are presented as mean ± SD. Categorical variables are given as frequencies and percentages. The results of the qualitative analyses were compared using Friedman’s ANOVA. For the CV comparisons, a repeated measures ANOVA was conducted. All *p* values were corrected for multiple testing using the Bonferroni correction. *p* values < 0.05 were considered to indicate statistical significance.

## Results

In total, 48 patients were prospectively included for analysis. All reconstructions were successfully calculated for all patients.

The mean age was 66.5 ± 11.2 years, 50% (*n* = 24) were males, and the mean BMI was 25.2 ± 4.7 kg/m^2^. The majority of patients underwent CT for the clinical workup of oncological diseases. The mean CTDI_vol_ was 6.2 ± 6 mGy. Further details are presented in Table 1.

**Table 1:** Patient characteristics and radiation dose

Variable	*N* (%) or mean ± SD
Participants	48 (100%)
Sex (male)	24 (50%)
Age (years)	66.5 ± 11.2
Weight (kg)	74.5 ± 16.2
Height (m)	1.7 ± 0.08
BMI (kg/m^2^)	25.2 ± 4.7
Clinical diagnosis	
Lymphoma	11 (22.9%)
Multiple myeloma	17 (35.4%)
Head and neck cancer	10 (20.1%)
*- Squamous*	7 (14.5%)
*- Adeno*	3 (6.3%)
Melanoma	5 (10.4%)
Other	5 (10.4%)
Radiation dose	
*CTDI*_*vol*_* (mGy)*	6.2 ± 6

### Qualitative image analysis

**Fig 3:**
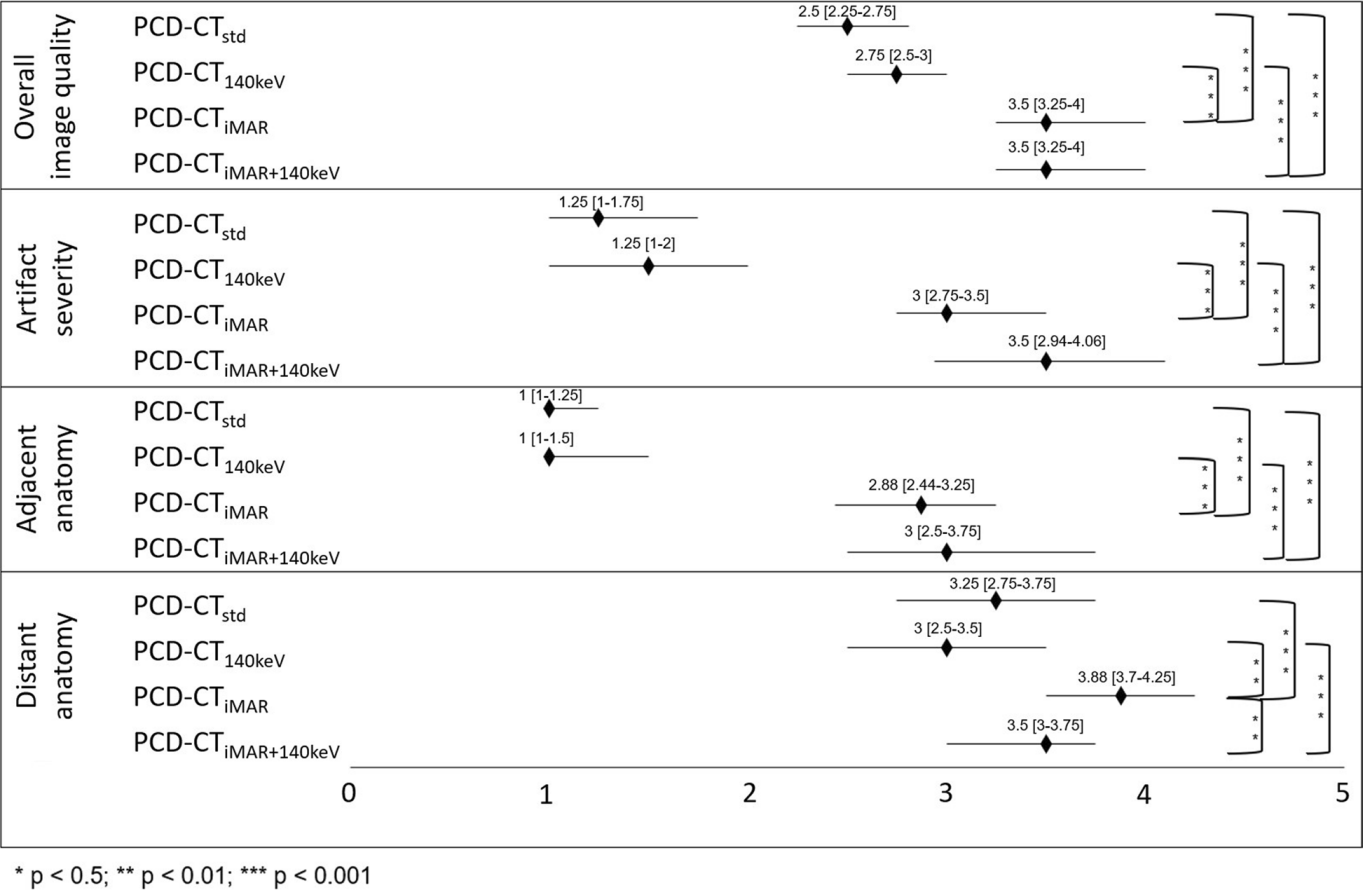
Results of the qualitative image analysis: Reading results are presented as median (black diamond) and interquartile ranges. *p* values are Bonferroni corrected for multiple comparisons. Nonsignificant comparisons are not indicated. PCD-CT_std_, standard reconstruction without metal artifact reduction; PCD-CT_140keV_, virtual monoenergetic reconstruction at 140 keV; PCD-CT_iMAR_, iterative metal artifact reduction algorithm; PCD-CT_iMAR+140keV_, iterative metal artifact reduction algorithm in combination with virtual monoenergetic reconstruction at 140 keV

All reading sessions were successfully completed by the four radiologists. Results of the qualitative image analyses are given in Fig. 3.

Friedman’s ANOVA yielded highly significant differences in overall image quality (Friedman’s *Q* (df = 3) 104; *p* < 0.001), artifact severity (Friedman’s *Q* (df = 3) 124.6; *p* < 0.001), and delineation of adjacent (Friedman’s *Q* (df = 3) 124.1; *p* < 0.001) as well as distant anatomy (Friedman’s *Q* (df = 3) 61.2; *p* < 0.001). The highest overall image quality was observed for PCD-CT_iMAR+140keV_ (3.5 [3.3–4]) and PCD-CT_iMAR_ (3.5 [3.3–4]). Lowest ratings were found for PCD-CT_std_ (2.5 [2.3–2.6]). A post hoc pairwise comparison employing Bonferroni correction revealed significant differences between the non-iMAR and the iMAR reconstructions (*p* < 0.001). Similar results were found for artifact severity and adjacent anatomy (all *p* values < 0.001). For delineation of distant anatomy, the highest reading scores were observed for PCD-CT_iMAR_ (3.9 [3.5–4.3]), while the lowest ratings were found for PCD-CT_140keV_ (3 [2.5–3.5]). In post hoc testing, significant differences were observed for PCD-CT_iMAR_ vs. PCD-CT_iMAR+140keV_, for PCD-CT_iMAR_ vs. PCD-CT_std_, and for PCD-CT_iMAR+140keV_ vs. PCD-CT_140keV_ (all *p* values < 0.01). A representative image example is provided in Fig. 2.

### Quantitative image analysis

#### Coefficient of variation

**Fig 4:**
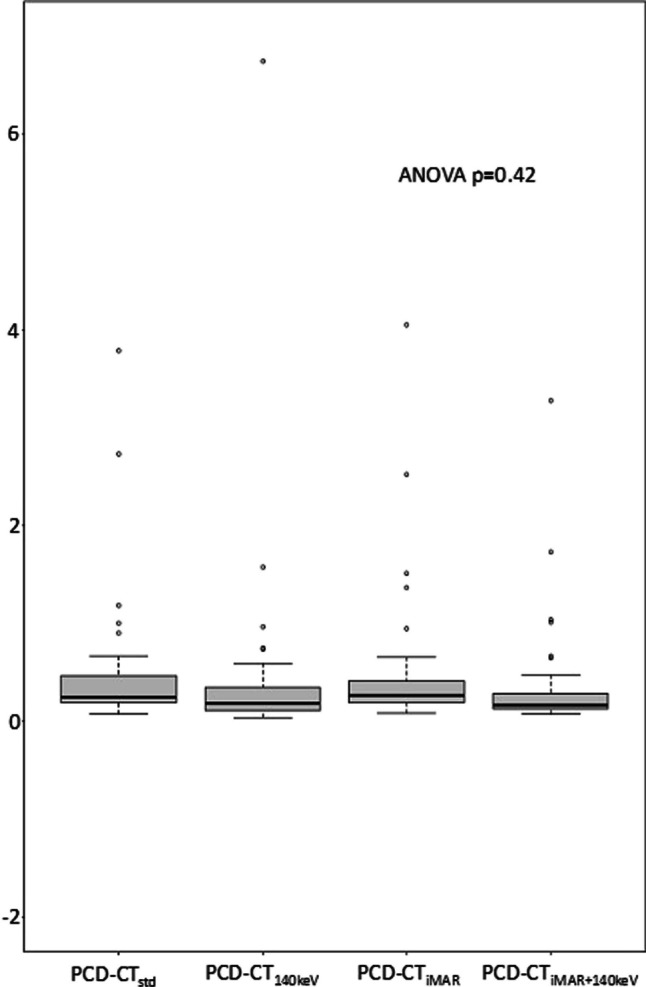
Boxplots displaying the results of the coefficient of variation analysis as a proxy for signal homogeneity measured in muscle tissue visually not affected by the artifact. ANOVA did not yield significant results (*p* = 0.42). PCD-CT_std_, standard reconstruction without metal artifact reduction; PCD-CT_140keV_, virtual monoenergetic reconstruction at 140 keV; PCD-CT_iMAR_, iterative metal artifact reduction algorithm; PCD-CT_iMAR+140keV_, iterative metal artifact reduction algorithm in combination with virtual monoenergetic reconstruction at 140 keV

Mean CV values for the different reconstructions were as follows: PCD-CT_std_ = 0.45 ± 0.64, PCD-CT_140keV_ = 0.40 ± 0.97, PCD-CT_iMAR+140keV_ = 0.34 ± 0.52, and PCD-CT_iMAR_ = 0.48 ± 0.67 (see Fig. 4). Repeated measures ANOVA of the CV analysis revealed no significant effect of the artifact reduction approach on image signal homogeneity (*p* = 0.42).

#### Quantification of artifact reduction

**Fig 5:**
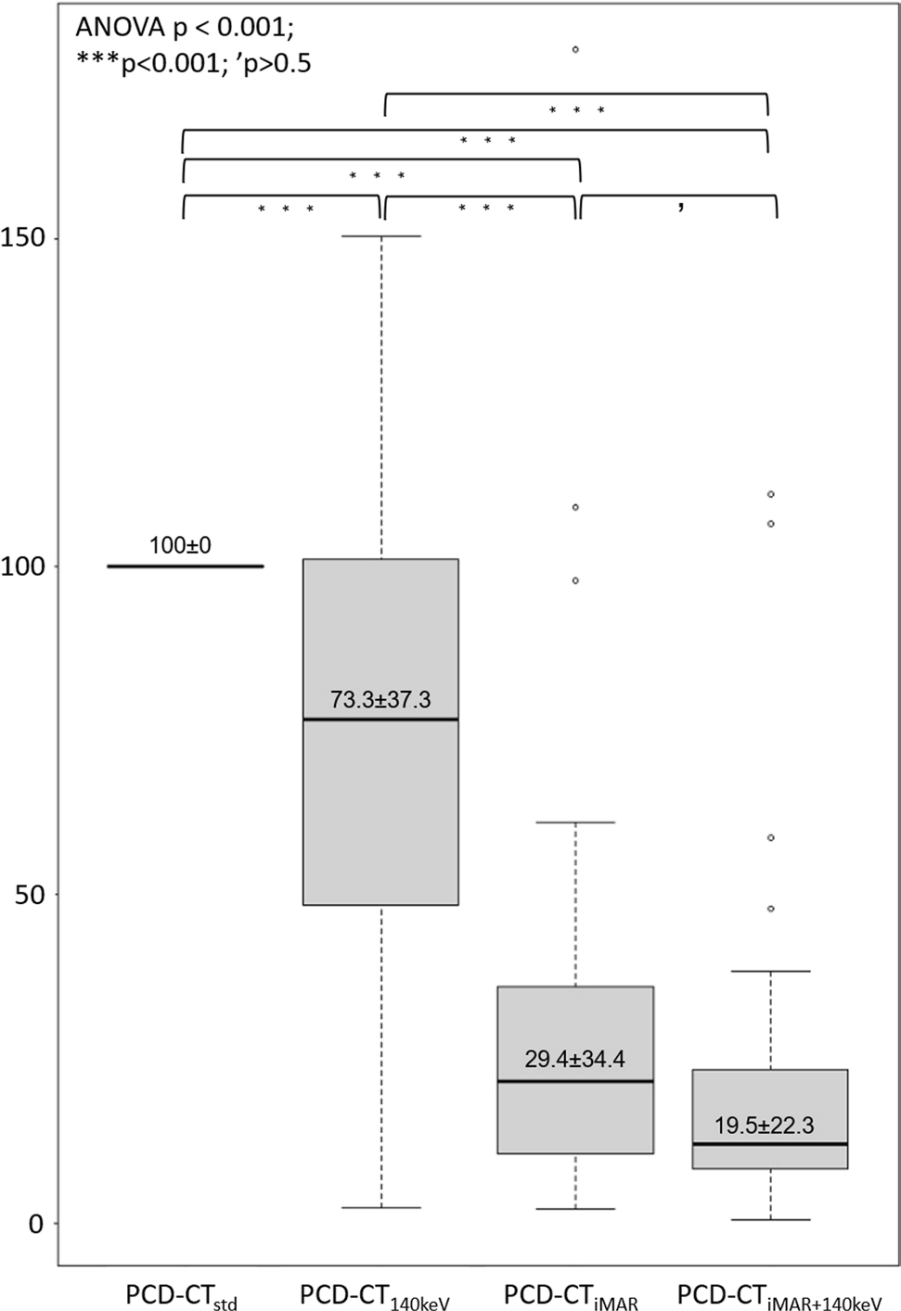
Boxplots displaying the results of the quantitative artifact reduction analysis. To objectively quantify and compare the artifact reduction potential of the different investigated artifact reduction approaches, we estimated the change in artifact severity based on relative changes in Hounsfield units (HU) within the artifact. Pairwise comparisons revealed significant differences between the artifact reduction techniques except for PCD-CT_iMAR_ vs. PCT-CT_iMAR+140keV_. PCD-CT_std_, standard reconstruction without metal artifact reduction; PCD-CT_140keV_, virtual monoenergetic reconstruction at 140 keV; PCD-CT_iMAR_, iterative metal artifact reduction algorithm; PCD-CT_iMAR+140keV_, iterative metal artifact reduction algorithm in combination with virtual monoenergetic reconstruction at 140 keV; ROI, region of interest; HU, Hounsfield units

The mean quotient of the reconstructions was as follows: PCD-CT_std_ = 100 ± 0, PCD-CT_140keV_ = 73.3 ± 37.3, PCD-CT_iMAR+140keV_ = 19.6 ± 22.3, and PCD-CT_iMAR_ = 29.4 ± 31.4. Repeated measures ANOVA for the ROI-based artifact reduction analysis indicated a significant impact of the different artifact reduction techniques on artifacts severity (*F* value = 114.5, df = 2 (*p* < 0.001)). Post hoc pairwise comparisons (Bonferroni corrected) yielded highly significant differences for iMAR vs. non-iMAR reconstructions (*p* < 0.001). No significant difference was found for PCD-CT_iMAR_ vs. PCD-CT_iMAR+140keV_; *p* = 0.2) (Fig. 5).

## Discussion

In this study, we investigated the impact of different metal artifact reduction methods in patients with dental hardware using a first-generation PCD-CT system. We found the most effective artifact reduction for the iMAR algorithm alone and in combination with virtual monoenergetic images at 140 keV, whereas the 140 keV reconstructions alone revealed only a moderate performance.

Our findings are of clinical importance, as artifacts caused by metallic implants remain a major challenge in CT imaging, with substantial impairment of image quality posing the risk of missing relevant findings [6, 12]. Our results demonstrate that PCD-CT with iMAR post-processing allows for significant artifact reduction and improvement of subjective and objective image quality with no additional scan or reconstruction time. This yields the potential to identify pathologies (e.g., fractures or abscesses) in regions previously obscured by artifacts and thus improve patient management.

Our subjective image analysis revealed the most effective artifact reduction and superior assessment of anatomical structures (adjacent and distant) for PCD-CT_iMAR_ algorithm. For PCD-CT_iMAR+140keV_, we also observed effective artifact reduction, though ratings for distant anatomy were significantly lower compared to PCT-CT_iMAR_, most likely due to the reduced soft tissue contrast, which is an inherent limitation of virtual monoenergetic images at high keV. The PCD-CT_140keV_ reconstruction revealed only moderately improved reading scores compared to the PCD-CT_std_ reconstruction but was significantly inferior compared to the two iMAR approaches. These results are corroborated by the quantitative analysis, where PCD-CT_iMAR_ and PCD-CT_iMAR+140keV_ resulted in the most effective artifact reduction whereas only a moderate effect was found for PCD-CT_140keV_. These findings are in line with previously published literature. For example, we used a second-generation dual-source scanner to investigate iMAR algorithms in 30 patients with dental hardware. Here, a significant reduction in metal artifact was found leading to improved image quality and delineation of anatomy [23]. Another recent study on 44 patients investigated the impact of iMAR and virtual monoenergetic reconstructions with a split-beam single-source dual-energy EID-CT. Again, a superior artifact reduction for the iMAR algorithms was observed and similar non-significant results for the combination of virtual monoenergetic reconstruction with iMAR compared to iMAR alone were reported [24].

Image post-processing always comes at the risk of altering the image signal and information with potentially unpredictable consequences for subsequent analyses. This might be of interest when evaluating the iodine uptake of a lesion to differentiate benign from potentially malignant findings. Our CV analysis indicated that the applied artifact reduction approaches did not affect the overall signal homogeneity as no significant differences in CV were observed between the different reconstructions.

Various artifact reduction techniques have already been investigated for conventional energy-integrating CT systems and have shown similar beneficial results for several types of foreign materials such as dental hardware, hip implants, and dorsal instrumentation [7, 12, 23]. This does not only apply for iMAR techniques but also for high keV monoenergetic reconstructions that up until now could only be calculated with dual-energy EID-CT systems [9, 10, 25]. With the novel photon-counting detector technology, multispectral data is intrinsically acquired in every scan and can be used for advanced post-processing (iMAR and high keV monoenergetic reconstructions) whenever clinically necessary [13].

The following limitations need to be considered. First, we investigated a variety of different dental hardware (restorations, replacements, prostheses) without further information about the vendor and composition of the restorations and implants. Secondly, we included consecutive patients scheduled for a clinically indicated CT. Whether the different artifact reduction techniques allow for the detection of more clinically relevant findings than standard reconstruction and alter clinical decision-making needs to be investigated in more focused studies including patients with head and neck pathologies.

In conclusion, PCD-CT provides highly effective methods for metal artifact reduction and allows for a significant improvement of subjective and objective image quality with the potential to overcome current diagnostic challenges in patients with dental hardware.

## Abbreviations

CV: Coefficient of variation
EID: Energy-integrating detectors
iMAR: Iterative metal artifact reduction
PCD-CT: Photon-counting detector computed tomography

## Declarations

### Guarantor

The scientific guarantor of this publication is Jakob Weiss.

### Statistics and biometry

No complex statistical methods were necessary for this paper.

### Study subjects or cohorts overlap

Some study subjects or cohorts have not been previously reported.

### Methodology

• prospective

• experimental

• performed at one institution
